# Feasibility of a breath robot intervention to reduce sleep problems in posttraumatic stress disorder: A randomized controlled study

**DOI:** 10.1016/j.ijchp.2025.100594

**Published:** 2025-06-06

**Authors:** Annett Lotzin, Carlotta Reinhardt, Michael Barthelmäs, Isabelle Laskowsky

**Affiliations:** a Institute for Clinical Psychology and Psychotherapy, MSH Medical School Hamburg, Hamburg, Germany; b Department of Psychiatry and Psychotherapy, University Medical Center Hamburg-Eppendorf, Hamburg, Germany; c Department of Social Psychology, University of Ulm, Ulm, Germany

**Keywords:** Posttraumatic stress disorder, Breath robot, Computer-human interaction, Sleep problems, Stress symptoms, Randomized controlled trial, Feasibility study

## Abstract

Background: Patients with Posttraumatic stress disorder (PTSD) suffer from sleep problems. Robot interventions offer a novel approach to reducing these problems. However, it is unclear whether patients with PTSD accept and regularly use a robot intervention and whether it is effective. Objective: This randomized controlled study evaluated the feasibility of a novel breath robot intervention in PTSD patients. Methods: Thirty-one adults with PTSD according to DSM-V (PSSI-5) with impaired sleep (PSQI > 5) were randomly assigned to a 4-week robot intervention (Somnox 2) with human breath simulation or to a 4-week robot intervention without human breath simulation. The primary feasibility outcome was the proportion of randomized participants providing outcome data at post-treatment. Secondary feasibility outcomes involved eligibility rate, recruitment speed, uptake, retention, treatment adherence, and dropout, clinical outcomes included sleep quality (PSQI), PTSD symptoms (PSSI-5), distress (PSS-10), and well-being (WHO-5). Results: Outcome data were provided by 96.8 %; eligibility rate was 75.6 %; 31 patients were recruited within 6 months; uptake and retention were 100 %; treatment adherence was 93.8 % in the intervention and 92.9 % in the active control group; dropout was 0 %. Distress decreased significantly more in the intervention group compared to the active control group (*SMD =* 0.67, 95 % CI 0.39, 0.94), but sleep quality, PTSD symptoms, and well-being did not improve significantly more. Conclusions: The use of a 4-week breath robot intervention was feasible and highly accepted in patients with severe PTSD. The breath robot intervention may reduce distress but may not be superior to a robot intervention without breath simulation for improving sleep and PTSD symptoms. Future trials should further determine the intervention’s clinical benefit.

## Introduction

Posttraumatic stress disorder (PTSD) is characterized by re-experiencing memories of a traumatic event, avoidance of trauma-related stimuli, and psychophysiological hyperarousal (American Psychiatric Association, 2013). Eight out of ten PTSD patients report difficulties falling or staying asleep (Colvonen et al., 2018). Although sleep problems impair quality of life, they are often unaddressed in PTSD treatment (Dai et al., 2019; Schubert et al., 2002).

Slow breathing techniques can reduce sleep problems (Laborde et al., 2019) by parasympathetic activation (Zaccaro et al., 2018) and reduction in distress and arousal (Zaccaro et al., 2018). While psychotherapists have applied breathing practices in PTSD patients to promote relaxation (Brown et al., 2013), the application of a robot intervention simulating human breath to reduce sleep problems in PTSD is a novel approach that has not been tested.

To date, robots that interact with humans have rarely been used to treat mental disorders. The first applications concern their use to enhance social interaction in adults with dementia (Góngora Alonso et al., 2019; Valentí Soler et al., 2015), and to support children with autism spectrum disorders to recognize and respond appropriately to emotions (Pennisi et al., 2016). If effective, robot interventions could be used to supplement mental health treatment or to bridge waiting times. Compared to traditional treatment, robot interventions require less time, space, and staff and can be used in the home environment. However, it remains unclear whether patients with PTSD accept robot interventions and regularly engage with them.

A robot intervention applying the mechanism of breath slowing is a novel approach to address the treatment gap regarding sleep problems in PTSD. A 2-week robot intervention with human breath simulation has been examined in *N*  =  36 premature infants to improve sleep (Ingersoll & Thoman, 1994). The breathing teddy bear robot adapted its breath frequency to the infant when in contact, which improved sleep post-intervention. A pilot study in *N* = 9 girls aged 8 to 10 years exposed to disasters found that a teddy bear breath robot intervention lowered the child’s breath rate compared to a robot intervention with deactivated breath function (Uratani & Ohsuga, 2018). A breath robot simulation intervention in *n* = 22 adult anxiety disorder patients and *n* = 21 healthy controls was evaluated positively, all participants used the robot continuously, and anxiety was reduced in the anxiety group (Matheus et al., 2022).

Somnox is a breath robot simulating human breath to reduce sleep problems. When being hugged, the robot records the human breath and simulates human breathing at a slightly slower pace. A pilot RCT examined the effects of a 3-week Somnox intervention in *N = *44 healthy adults with sleep problems (Støre, Tillfors, Wästlund et al., 2023). Adherence to the intervention was below 50 % and no reduction in insomnia symptoms was found. In contrast, a 2-week Somnox intervention in four seniors with sleep problems reported a significant increase in sleep duration (Rădulescu, 2024). A feasibility study in four insomnia patients found that the Somnox intervention was safe and accepted, and sleep problems were reduced in some but not all participants. Another pilot study on the Somnox intervention in four patients with attention deficit hyperactivity disorder with insomnia problems reported inconsistent effects on insomnia symptoms (Støre, Tillfors, Angelhoff et al., 2023). Given the small sample sizes, and the heterogeneous populations and results of the previous research (Rădulescu, 2024; Støre, Tillfors, Angelhoff et al., 2023, 2023), more evidence is needed to evaluate the feasibility and efficacy of the Somnox intervention to reduce sleep problems. No study so far has evaluated the feasibility and effectiveness of a breath robot intervention to reduce sleep problems in PTSD.

The primary aim of this study was to investigate the feasibility of a novel breath robot intervention (Somnox) in patients with PTSD. The secondary aim was to assess preliminary data on the intervention’s effectiveness in reducing sleep problems and PTSD symptoms.

## Methods

### Design

We employed an evaluator-blinded randomized controlled pre-post feasibility study. Participants were randomly allocated to a 4-week robot intervention with human breath simulation or to a robot control group without human breath simulation. The study was preregistered (German Clinical Trials Register, DRKS00031063), and the procedure was published in a study protocol (Lotzin & Laskowsky, 2024). The Ethics Committee at MSH Medical School Hamburg provided ethical approval (MSH-2 022/200). The manuscript was prepared according to the CONSORT guidelines for reporting pilot and feasibility studies (Eldridge et al., 2016).

### Study setting

Individuals with PTSD were recruited from the universities’ psychotherapeutic outpatient unit and through advertisements on websites such as eBay Classified Ads.

### Sample

Inclusion criteria: (1) aged 18 years or older; (2) diagnosed with at least subsyndromal PTSD (trauma exposure, total score ≥ 23 in the PTSD Symptom Scale – Interview for DSM-5, PSSI-5 (Foa et al., 2016), at least one intrusion, and one arousal symptom according to PSSI-5); (3) impaired sleep quality (Pittsburgh Sleep Quality Index, PSQI (Buysse et al., 1989) > 5). Exclusion criteria: (1) organic sleep disorder (e.g., sleep apnea, narcolepsy, restless legs syndrome); (2) psychotherapeutic treatment during the study period, except for initial psychological consultations or probationary sessions for treatment planning. The participants also had to agree to use the robot every day and to document the use of the robot and their sleeping habits daily via smartphone.

### Intervention

**Fig. 1:**
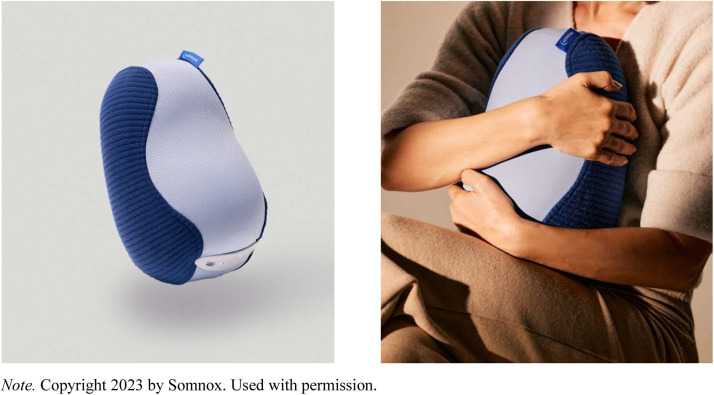
Somnox 2 breath robot Note. Copyright 2023 by Somnox. Used with permission.

The Somnox robot was developed at the Robotics Institute of Delft University of Technology, Netherlands to improve sleep quality by slowing down breathing (van Oers & Stoevelaar, 2019). The latest version of the robot (Somnox 2) takes the form of a bean-shaped cushion with a soft surface pleasant to touch (Fig. 1). When embraced, the robot senses the user's breathing rhythm and simulates a human breath at a slightly slower rate. Participants of the intervention group received a Somnox 2 robot simulating human breathing. Participants of the active control group received the same robot with a deactivated breathing function. Over the 4-week study period, all participants were instructed to hold the robot in their arms for a minimum of 30 min. each day in bed until they fell asleep and to resume this practice if they woke up during the night. They could also use the robot throughout the day.

#### Feasibility outcomes

Main outcome: proportion of randomized participants providing outcome data at post-treatment (T1). We considered a proportion of> 50 % to indicate feasibility. The following feasibility outcomes were defined as secondary outcomes:

1. *Eligibility rate* (proportion of included participants out of all screened individuals)
2. *Recruitment speed* (number of participants recruited in 6 months)
3. *Uptake* (proportion of participants who started the intervention out of those randomized in the intervention group)
4. *Retention* (proportion of participants that received at least 20 robot sessions)
5. *Treatment adherence* (proportion of days of robot use, average daily robot use in minutes)
6. *Drop-out rate* (proportion of randomized participants who terminated the study before T1 completion)

#### Clinical outcomes

To gain first insights into the effectiveness of the intervention in patients with PTSD, we assessed the following secondary clinical outcomes: sleep quality (Pittsburgh Sleep Quality Index, PSQI; Buysse et al., 1989), PTSD symptom severity (PTSD Symptom Scale - Interview for DSM-5, PSSI-5; Foa et al., 2016), perceived distress (Perceived Stress Scale-10, PSS-10; Schneider et al., 2020), and well-being (World Health Organization Well-Being Index, WHO-5; Topp et al., 2015) were assessed before (T0) and after (T1) the 4-week intervention.

### Measures

Sociodemographic and health-related characteristics were assessed at T0 using self-constructed items.

#### Clinical characteristics

Clinical symptoms were measured at T0 and T1. Sleep quality in the past two weeks was measured with the Pittsburgh Sleep Quality Index (Buysse et al., 1989). The PSQI consists of 19 items grouped into seven component scores (scored on 4-point scales from 0 to 3): subjective sleep quality, sleep latency, sleep duration, sleep efficiency, sleep disturbances, use of sleep medication, and daytime dysfunction. A total score ranging from 0 to 21 and severity categories (0–5 healthy sleep; 6–10 poor sleep; 11–21 potential chronic sleep disorder) can be calculated, higher scores indicate a poorer sleep quality. The PSQI has shown good reliability and validity across studies (Backhaus et al., 2002; Buysse et al., 1989).

PTSD symptoms were measured using the 24-item semi-structured PTSD Symptom Scale – Interview for DSM-5 (PSSI-5; Foa et al., 2016). The PSSI-5 assesses a PTSD diagnosis and symptom severity according to DSM-5. The interview assesses symptoms of re-experiencing, avoidance, changes in cognition and mood, increased arousal and reactivity, and distress and interference. Item scores range from 0 (not at all) to 4 (6 or more times a week/severe) and refer to the past month. A total symptom severity score and severity categories can be calculated (0–8 = minimal; 9–18 = mild; 19–30 = moderate; 31–45 = severe; 46–80 = very severe). The interviews were conducted by blinded and trained research assistants. The PSSI-5 demonstrated good validity and reliability in adults trauma survivors (Foa et al., 2016).

Distress was assessed using the Perceived Stress Scale-10 (Schneider et al., 2020). The ten PSS-10 items (scored on 5-point scales from 1 *never* to 5 *very often*) can be summarized to a total score, with higher scores indicating higher perceived distress. The PSS-10 has shown good reliability and validity in adult populations (Lee, 2012).

Well-being was measured by the World Health Organization Well-Being Index (WHO-5; Topp et al., 2015). The WHO-5 includes five items (scored on 6-point scales from 0 *not present* to 5 *constantly present*) measuring psychological well-being. The WHO-5 has been shown reliability and validity in heterogenous settings (Sischka et al., 2020; Topp et al., 2015).

### Sample size and randomization

No formal sample size calculation was conducted as sample size calculations are not recommended for feasibility studies (Lancaster et al., 2004). *N* = 31 participants were randomly allocated to the robot intervention with breath simulation, or to the robot intervention without breath simulation. The allocation schedule for the random assignment to the two conditions (1:1 ratio, block size 5; DatInf RandList Version 1.2) was generated by a researcher uninvolved in other study procedures. The random numbers were stored in separate sequentially numbered opaque envelopes to conceal allocation until interventions were assigned.

### Procedure

The eligibility assessment was conducted at MSH Medical School Hamburg, Germany between February and August 2023. The research personnel informed about the study and its procedures and addressed queries. Each potential participant received a study sheet outlining the study's objectives, procedures, eligibility criteria, the random allocation process, study benefits and risks, compensation for participation, the right to withdraw from the study, data storage, and contact details. Potential participants underwent a 2-hour face-to-face baseline assessment (T0) to assess eligibility criteria and collect baseline data. Eligible individuals who agreed to participate in the study signed the informed consent form. The research staff instructed participants on how to use the breath robot and operate the technical equipment.

Over the 4-week study period, all participants were instructed to use the robot daily at home (see intervention section) and to record their use of the robot and sleep characteristics via a smartphone-based diary within 1h of their wake-up time. The link to the diary was delivered via Survey-Bot (Barthelmäs et al., 2021). A research assistant conducted weekly phone-based check-ins to offer technical support and address queries. After completion of the intervention (T1), participants attended another 2-hour face-to-face assessment to reassess PTSD symptoms using the PSSI-5. Participants also completed an online questionnaire at T0 and T1 to evaluate self-reported sleep quality, distress, and well-being.

### Statistical analysis

Descriptive statistics (means and standard deviations for continuous variables; absolute and relative frequencies for categorical variables) were computed for all outcomes (R Core Team, 2023), separately for the two groups if appropriate. Controlled effect sizes (Standardized Mean Difference, *SMD*) and their confidence intervals as well as pre-post effect sizes were computed per group for clinical outcomes (Morris, 2008).

## Results

### Participant flow

**Fig. 2:**
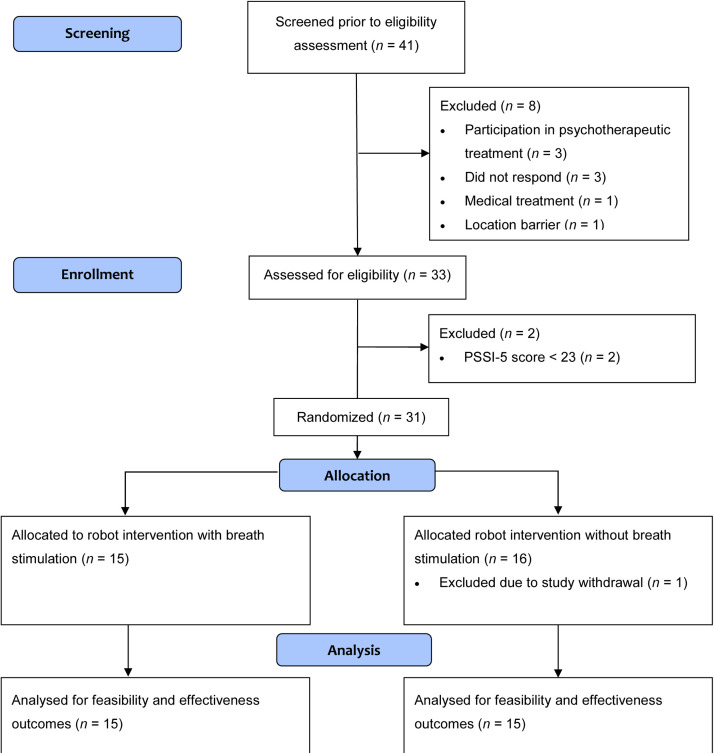
CONSORT flowchart of participants.

We pre-screened 41 individuals of which 8 were excluded (Fig. 2); 33 individuals underwent full examination of all eligibility criteria. *N* = 30 participants were randomized. One person withdrew study participation after completion of all study assessments, resulting in the deletion of the corresponding data and exclusion from analysis. An additional individual was included, resulting in *N* = 31 randomized participants. The final analysis set consisted of *N* = 30 participants.

### Sample characteristics

The sample composed individuals with varying age (intervention: *M* = 42.3 years; *SD* = 13.5; *Min* = 26; *Max* = 65; control *M* = 32 years; *SD* = 14.1; *Min* = 19; *Max* = 66) and income (Table 1). Most were female. All participants fulfilled the full diagnostic criteria for PTSD, most had severe or very severe PTSD (intervention 93.3 %, control 86.7 %; Table 2). In both groups, one out of two reported sexual abuse as index trauma.

**Table 1:** Sociodemographic characteristics (*N* = 30).

Variable	Intervention group (*n* = 15)		Control group (*n* = 15)	
	*n*	%	*n*	%
Gender				
Female	14	93.3	10	66.7
Male	1	6.7	4	26.7
Diverse	0	0.0	1	6.6
Marital status				
Unmarried	10	66.7	11	73.4
Married	3	20.0	2	13.3
Divorced	2	13.3	2	13.3
Partnership				
Single	6	40.0	10	66.7
Temporary relationship(s)	0	0.0	1	6.7
Relationship, living together	6	40.0	2	13.3
Relationship, living apart	3	20.0	2	13.3
Children				
Yes	5	33.3	3	20.0
Living situation				
Alone	5	33.3	8	53.4
With partner	6	40.0	2	13.3
With children	2	13.3	1	6.7
With parents	0	0.0	2	13.3
With friends	1	6.7	2	13.3
With others	1	6.7	0	0.0
Education level^a^				
Lower secondary education	9	60.0	6	40.0
High school diploma	5	33.3	7	46.7
University degree	1	6.7	2	13.3
Work status				
In training	1	6.7	3	20.0
Not working	8	53.3	5	33.3
Part-time	4	26.7	4	26.7
Full-time	2	13.3	3	20.0
Household income after taxes				
<1000€	1	6.7	3	20.0
1000€ to <2000€	6	40.0	7	26.6
2000€ to <3000€	2	13.3	1	6.7
3000€ to <5000€	3	20.0	1	6.7
Don’t want to report	3	20.0	3	20.0

Notes.

a Categories according to the International Standard Classification of Education (2012).

**Table 2:** Clinical characteristics (*N* = 30).

Variable	Intervention group (*n* = 15)	Control group (*n* = 15)	Intervention group (*n* = 15)	Control group (*n* = 15)
	*n*	*%*	*n*	*%*
PTSD diagnosis (PSSI-5)^a^				
Subsyndromal PTSD	0	0.0	0	0.0
Full PTSD	15	100.0	15	100.0
PTSD severity (PSSI-5) ^b^				
Moderate	1	6.7	2	13.3
Severe	11	73.3	4	26.7
Very severe	3	20.0	9	60.0
Type of Trauma (PSSI-5) ^c^				
Actual or threatened death	10	66.7	8	53.3
Actual or threatened serious injury	11	73.3	9	60.0
Actual or threatened sexual violation	8	53.3	7	46.7
Comorbid mental disorder in addition to PTSD				
Yes	12	80.0	10	66.7
Past psychiatric or psychotherapeutic treatment				
Yes	10	66.7	14	93.3
Type of past treatment ^d^				
Outpatient	8	53.3	10	66.7
Day clinic	5	33.3	5	33.3
Inpatient	8	53.3	10	66.7
				

Notes.

a PSSI-5 = PTSD Symptom Scale – Interview for DSM-5. ^c^ Scores of 19–30 indicate moderate, 31–45 severe, and 46–80 very severe severity. ^d^ Multiple answers possible.

### Feasibility outcomes

*Primary outcome:* Out of the *N* = 31 randomized participants, all (100 %) were reassessed at T1 (Table 3), but one participant withdrew from study participation. *N* = 30 participants 96.8 % provided outcome data at T1.

**Table 3:** Feasibility outcomes.

Outcome	Result
Reassessment	Out of the *N* = 31 randomized participants, all (100.0 %) were reassessed at post-treatment.
Eligibility	75.6 % (31 out of 41 screened) included.
Recruitment speed	*N* = 31 eligible participants were recruited within 6 months (175 days, 5.6 days per participant).
Uptake	100.0 % (15/15) of the intervention and 93.8 % (15/16) of the control group started the intervention.
Retention	100.0 % 815/15) of the intervention and 100.0 % (16/16) of the control group used the robot in at least 20 robot sessions.
Treatment adherence	Average proportion of days of robot use: intervention 93.8 %, control 92.9 %. Average daily robot use in min.: intervention *M* = 26.3 sessions (*Min =* 21, *Max* = 28), control *M* = 26.0 sessions (*Min* = 25, *Max* = 28).
Drop-out rate	All randomized participants completed the study. One participant withdrew study participation at a later timepoint.

### Secondary feasibility outcomes

Out of the 41 screened participants, 31 (75.6 %) met the eligibility criteria (Table 3). The most often exclusion reasons were ongoing psychotherapeutic treatment or loss of contact. *N* = 31 eligible participants were recruited within 6 months, most via announcements on websites (48.4 %) or via psychotherapeutic outpatient units (38.7 %). To recruit one participant, 5.6 days were needed, on average. All participants (100 %) started the robot intervention. All participants 100 % completed at least 20 robot sessions. Treatment adherence *was high:* Participants of the intervention group used the robot for 93.8 % of the 28 days, 53.5 min per day, on average. Participants of the control group used the robot for 92.9 % of the 28 days, 69.4 min per day, on average. No participant dropped out of the study within the study period.

### Clinical outcomes

**Fig. 3:**
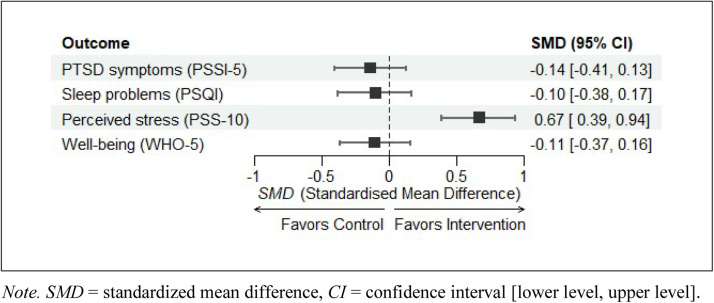
Standardized mean differences in the intervention vs. control group Note. SMD = standardized mean difference, CI = confidence interval [lower level, upper level].

Between-group *SMD* (Fig. 3) indicated that perceived distress decreased more greatly (medium effect size (Cohen, 1988)) in the intervention group than in the active control group (Fig. 3). No significant between-group differences were found in sleep quality, PTSD symptoms, and well-being.

All clinical outcomes improved in the intervention and active control group from T0 to T1 (Table 4), and pre-post effect sizes ranged from medium to large. Perceived distress decreased in both groups, with a significantly greater pre-post reduction (large effect size) in the intervention compared to the control group (medium effect size). Sleep problems decreased (large effect size) in both groups from "potential chronic sleep disorder" to "poor sleep", on average. PTSD symptoms decreased (medium effect size) in both groups but remained in the “severe” range. Well-being increased similarly (medium effect size) in both groups.

**Table 4:** Clinical symptoms and well-being at baseline and post-treatment in the intervention and control group (*N* = 30).

	Intervention group						Control group					
	T0		T1				T0		T1			
	(*n* = 15)		(*n* = 15)				(*n* = 15)		(*n* = 15)			
Variable	*M*	*SD*	*M*	*SD*	*SMD*	95 % CI	M	*SD*	*M*	*SD*	*SMD*	*95 % CI*
PTBS symptoms (PSSI-5)	40.4	8.5	34.5	11.5	−0.56	[−0.75, −0.38]	43.1	10.8	34.6	12.8	−0.70	[−0.89, −0.51]
Sleep problems (PSQI)	12.8	3.1	9.8	3.5	−0.88	[−1.07, −0.68]	11.8	3.0	8.5	3.5	−0.98	[−1.17, −0.78]
Distress (PSS-10)	37.9	5.6	31.6	4.4	−1.22	[−1.42, −1.02]	34.6	6.4	31.1	5.7	−0.56	[−0.75, −0.37]
Well-being (WHO-5)	5.3	2.7	7.7	4.4	0.65	[.46, 0.84]	6.1	2.9	8.6	5.7	0.54	[.35, 0.73]

Note. T0 = baseline, T1 = post-treatment, *SMD* = pre-post standardized mean difference, *CI* = confidence interval [lower level, upper level].

## Discussion

This feasibility study extends previous robotic mental healthcare research by focusing on a breath robot to reduce sleep problems in PTSD, an area that has not been previously explored. Within a randomized controlled study, we compared the feasibility of a 4-week robot intervention (Somnox 2) with human breath simulation to reduce sleep problems in PTSD, compared to a robot intervention without breath simulation. Preliminary data on clinical improvements due to the robot intervention were also assessed.

### Feasibility

The implementation of the 4-week breath robot intervention within an RCT was very feasible. Three-quarters of the screened participants were eligible for the study and the PTSD patients could be recruited within 6 months. Treatment adherence was high, the participants used the robot consistently on most days and used the robot longer than the recommended daily 30 min. We found a high participant retention, with all participants completing at least 20 out of 28 robot sessions. There were no dropouts observed throughout the study period, indicating high adherence to the intervention. These results indicate that the participants accepted the robot intervention and liked to use the robot before sleep. An earlier small feasibility study on the Somnox robot intervention in four insomnia patients also reported a high acceptance of the intervention. However, the high adherence found in our study contrasts with the findings of an earlier pilot RCT in healthy adults with sleep problems (Støre, Tillfors, Wästlund et al., 2023), which reported an adherence to the Somnox robot of below 50 %. Adherence to a breath robot intervention may be higher in severely ill patients with a high symptom burden, compared to a sample of healthy adults. Our results emphasize the feasibility of a breath robot intervention in patients with severe PTSD, highlighting the potential of robot interventions for successful implementation in future research and clinical practice in this patient group.

### Clinical effectiveness

Distress more greatly decreased in the breath robot intervention group compared to the robot control group without breath function. This finding aligns with previous research showing that slow breathing training was associated with distress reduction (Bernardi et al., 2001). However, sleep quality, PTSD symptoms, and well-being did not differ between the two active groups, both the intervention and control group showed small to large pre-post improvements. As most clinical improvements were found in both groups, they cannot be attributed to the breath function of the robot. This finding contrasts our assumption that the robot’s breath function may reduce sleep problems and PTSD symptoms and may limit its clinical value. Earlier studies on the Somnox breath robot intervention reported heterogeneous findings but did not target patients with PTSD, had small sample sizes, and did not include an active control group (Rădulescu, 2024; Støre, Tillfors, Angelhoff et al., 2023, 2023), making it difficult to compare the results. Future studies may examine whether the assumed mechanism of action, i.e., slowing the breath rate of the user, can be achieved by the intervention, which we did not directly test in this study.

Participants of the control group daily used the robot without breath function before sleep and showed similar improvements in sleep quality, suggesting mechanisms independent from the robot’s breath function. The Somnox’ soft shape and surface may have elicited feelings of comfort and safety which may promote relaxation and better sleep. Interacting visually and tacitly with a soft cuddly toy induced relaxation (Tribot et al., 2024) and reduced distress in earlier studies (Tai et al., 2011), and animal-like robots enhanced happiness and lowered oxytocin levels through tactile interaction (Geva et al., 2020). Establishing a routine before sleep with the regular use of the robot could also have improved sleep, as establishing a sleep routine is an effective element of sleep disorder behavioral therapy (Irish et al., 2015; Posner & Gehrman, 2011).

### Strengths and limitations

The study provides insights into the feasibility of a novel breath robot intervention implemented within a randomized controlled trial, the desired design for larger studies. We recruited a sample including low-income and low-educated participants, an underrepresented group in clinical research, thereby enhancing the generalizability of the findings. We diagnosed PTSD using a structured clinical interview and used validated self-report measures for clinical outcomes. We compared the breath robot intervention with an active robot control group. Such a study design can disentangle the effects of the breath function from the effects caused by other intervention features. While this approach broadens the understanding of the effects of the breath function, the study cannot provide information on whether the complete robot intervention, including hugging the robot daily with a pleasant texture and listening to the robot's breathing, reduces sleep problems in PTSD compared to no intervention. Future studies may include both an active control and a waitlist control group to compare reductions in PTSD between these groups.

The study was designed to test feasibility but not effectiveness; the small sample size constrains final conclusions regarding the intervention’s effectiveness on clinical symptoms. The absence of a follow-up assessment precludes determining the long-term sustainability of the observed improvements.

### Practical and research implications

Robot interventions hold promise for enhancing mental health treatment. They require less time, space, and staff compared to human treatment, which seems important in times of financial and professional shortages in the healthcare system. Robot interventions could bridge waiting times for psychological treatment and complement psychotherapeutic treatment to enhance their effectiveness. According to our findings, patients with PTSD accept a breath robot intervention and regularly engage with it, a requirement for implementation in healthcare. Moving forward, research should focus on understanding the mechanism of action underlying a breath robot intervention to optimize its efficacy and integration into clinical practice.

## Conclusions

A 4-week robot intervention with breath simulation was feasible and highly accepted in patients with severe PTSD. The intervention may reduce distress but may not be superior to a robot intervention without breath simulation for improving sleep and PTSD symptoms. Future efficacy trials should determine the clinical benefit of the breath robot intervention. If effective, the robot intervention may have the potential to improve mental health in PTSD patients.

## Conflicts of interest

All authors declare that they have no conflicts of interest which might have influenced the research reported in this paper.

## Data availability

The data used in this manuscript may be made available to researchers in anonymized form by the first author.

## CRediT authorship contribution statement

**Annett Lotzin:** Conceptualization, Methodology, Funding acquisition, Supervision, Formal analysis, Writing – original draft, Writing – review & editing. **Carlotta Reinhardt:** Data curation, Writing – review & editing. **Michael Barthelmäs:** Software, Writing – review & editing. **Isabelle Laskowsky:** Project administration, Data curation, Formal analysis, Writing – review & editing.

## Declaration of competing interest

The authors declare the following financial interests/personal relationships which may be considered as potential competing interests:

Annett Lotzin reports equipment, drugs, or supplies was provided by provision of the robots used in this study. Annett Lotzin reports a relationship with Daimler and Benz Foundation that includes: funding grants. If there are other authors, they declare that they have no known competing financial interests or personal relationships that could have appeared to influence the work reported in this paper.
